# Nutritional management and follow up of infants and children with food allergy: Italian Society of Pediatric Nutrition/Italian Society of Pediatric Allergy and Immunology Task Force Position Statement

**DOI:** 10.1186/1824-7288-40-1

**Published:** 2014-01-03

**Authors:** Marcello Giovannini, Enza D'Auria, Carlo Caffarelli, Elvira Verduci, Salvatore Barberi, Luciana Indinnimeo, Iride Dello Iacono, Alberto Martelli, Enrica Riva, Roberto Bernardini

**Affiliations:** 1 Department of Pediatrics, San Paolo Hospital, University of Milan, Milan, Italy; 2 Department of Pediatrics, University of Parma, Parma, Italy; 3 Department of Pediatrics, "Sapienza" University of Rome, Rome, Italy; 4 Department of Pediatrics, Fatebenefratelli Hospital, Benevento, Italy; 5 Department of Pediatrics, Guido Salvini Hospital, Garbagnate, Italy; 6 Department of Pediatrics, San Giuseppe Hospital, Empoli, Italy

**Keywords:** Food allergy, Nutritional status, Dietary intake, Cow's milk allergy, Follow-up

## Abstract

Although the guidelines on the diagnosis and treatment of food allergy recognize the role of nutrition, there is few literature on the practical issues concerning the nutritional management of children with food allergies.

This Consensus Position Statement focuses on the nutritional management and follow-up of infants and children with food allergy.

It provides practical advices for the management of children on exclusion diet and it represents an evidence-based consensus on nutritional intervention and follow-up of infants and children with food allergy.

Children with food allergies have poor growth compared to non-affected subjects directly proportional to the quantity of foods excluded and the duration of the diet. Nutritional intervention, if properly planned and properly monitored, has proven to be an effective mean to substantiate a recovery in growth.

Nutritional intervention depends on the subject’s nutritional status at the time of the diagnosis.

The assessment of the nutritional status of children with food allergies should follow a diagnostic pathway that involves a series of successive steps, beginning from the collection of a detailed diet-history.

It is essential that children following an exclusion diet are followed up regularly.

The periodic re-evaluation of the child is needed to assess the nutritional needs, changing with the age, and the compliance to the diet.

The follow- up plan should be established on the basis of the age of the child and following the growth pattern.

## Introduction

Currently guidelines establish that the treatment of food allergy (FA) consists of avoiding the food or foods that cause the symptoms. This is true for IgE mediated food allergies, non-IgE mediated, and the mixed forms IgE and non-IgE mediated [1-4].

To make certain that all the nutrients present in the food or foods excluded from the diet are provided from alternative sources, a plan of nutrition intervention must be arranged regardless of whether is for only a single food item or an entire food group. Appropriate nutritional indications must be provided to primarily ensure an adequate caloric intake, in addition to macro and micronutrients.

Furthermore, an appropriate follow- up plan is warranted to assess the compliance to the diet, identify early signs of nutritional deficiencies, and verify the development of tolerance.

Although the guidelines on the diagnosis and treatment of food allergy recognize the role of nutrition, there is few literature on the practical issues concerning the nutritional management of children with FA.

This position statement has been prepared by the joint Task Force of The Italian Society of Pediatric Nutrition (SINUPE) and the Italian Society of Pediatric Allergy and Immunology (SIAIP). This document represents a practical consensus on nutritional intervention in children with FA.

### Aim

This paper sets out to present nutritional issues in management and follow-up of food allergies.

The aims of the reading of the paper are as follows:

(i) Know the risk factors causing poor growth in children with food allergy

(ii) Assess the nutritional status of children with food allergy

(iii) Recognize early signs of nutritional deficiency

(iv) Prescribe an appropriate diet

(v) Set up an appropriate follow- up plan

### Growth and food allergy

Children with FA have a growth deficit compared to non-affected subjects [5-7] showing a positive correlation with the number of foods excluded and the duration of the diet [8,9].

The growth deficit is already evident during the first year of life regardless of the type of diet [7] and it can also be observed in allergic children with a caloric-proteic intake not different from healthy subjects [8]. Although the etiopathogenetic mechanisms are not entirely clear, several not mutually exclusive hypotheses have been called into question.

The state of inflammation that characterizes the allergic disease may result in reduced bioavailability of nutrients or an excessive loss of the same due to the increased intestinal permeability [10] caused by a poor compliance to the diet or by the presence of coexisting undiagnosed food allergies. Another possible explanation could be the increase of caloric needs, as shown indirectly by the effect of nutritional intervention on the anatomical and functional recovery of wound healing [11].

Some specific allergic conditions, such as eosinophilic esophagitis and eosinophilic gastroenteritis are often accompanied by loss of appetite and early satiety, which may further reduce the individual’s nutritional intake [12].

Finally, children on exclusion diet may develop dislike towards tolerated foods leading to further diet restrictions that can significantly contribute to a reduced intake of calories and nutrients [13,14].

There are many risk factors leading to the poor growth of children suffering from food allergies, summarized as follows (adapted from Venter) [15]:

(a) Delayed Diagnosis

(b) Onset of disease in early age

(c) Multiple food allergies

(d) Disease in active phase

(e) Persistent intestinal inflammation (subclinical)

(f) Elimination of most foods from the diet

(g) Elimination from the diet of foods with high nutritional value (milk, eggs)

(h) Poor compliance to dietary management (unwillingness to expand the diet)

(i) Extreme self-limitation of food

(j) Association with atopic diseases (asthma, atopic eczema) or with chronic diseases

Nutritional intervention, if properly planned and properly monitored, has proven to be an effective mean to substantiate a recovery in growth [16], which in turn is shown to correlate with the state of the individual’s health later on as an adult [17].

### Nutritional assessment

The objectives of nutritional intervention in patients are: 1. prevent allergic reactions, 2. ensure adequate growth and development, 3. recognize and treat malnutrition.

The family pediatrician must be familiar with the eating habits of the allergic child, closely monitor the growth, be able to recognize when the nutritional assessment is necessary and direct the child to specialist allergy clinics staffed by a range of health professionals who specialize in allergy (e.g. allergists, nutritionists, dietitians, clinical psychologists) for a proper dietary intervention and an appropriate follow- up plan.

The assessment of the nutritional status of children with FA should follow a diagnostic pathway that involves a series of successive steps: detailed diet history, assessment of growth, recording of nutritional intake and nutritional parameters, nutritional intervention and an appropriate follow-up.

#### 

##### Step 1. Obtaining a detailed diet history to identify dietary factors contributing to nutritional risk

**Keynote 1** The collection of a detailed diet history is essential for a proper evaluation. The information that must be obtained through a targeted diet history are summarized as follows:

(a) Type of infant feeding

(b) Age of solid food introduction and integration schedule of various solid foods

(c) Details on the foods excluded and the reasons for exclusion

(d) Type and quantity of water and drinks consumed

(e) Use of special formulas and daily intake during the day

(f) Intake of vitamin supplements and/or minerals, and dosage

(g) Aversion of certain foods

(h) Predilection of certain foods (from those permitted)

(i) Configuration of a typical meal

(j) Number of daily meals and distribution throughout the day

#### 

##### Step 2. Assessment of anthropometric measurements

The assessment of anthropometric measurements represents the most important step of nutritional assessment, because growth is a sensitive indicator of an adequate intake of energy and proteins. During the visit, weight, length, head circumference should be measured, and exclusively for children over 2 years of age, body mass index (BMI). The values must be reported on the appropriate growth curves.

For the first two years of life the World Health Organization (WHO) curves for weight and length by age, representing the reference standard [18,19] should be used. WHO growth curves are shown both in the form of percentiles and z-score: the normal range is between 2^nd^ and 98^th^ percentile which corresponds to approximately - 2 Standard Deviation (SD) and + 2 SD.

For older children (> 2 years) Centers for Diseases Controls and Prevention (CDC) growth charts should be used [20]: the normal limits are between the 5^nd^ and 95^th^ percentile for weight and height.

Children whose weight, length or height is below the 5^nd^ percentile or that experience a negative variation of two or more percentiles within a year are at nutritional risk and deserve a nutritional assessment.

For older children (> 2 years) the BMI curves [21] allow to diagnose a condition of pathological thinness and an underlying risk of malnutrition.

Even if single measurements of weight, height and the different ratios provide useful information, a more sensitive index of growth is growth velocity, the increase in height per unit of time. The slowing of the growth velocity is the most sensitive nutritional risk indicator supporting the need for a nutritional intake assessment [22]. In contrast, nutritional intervention is perceived in a "catch up" of the growth velocity, which is the most sensitive indicator for monitoring its effectiveness [23].

Currently, it is assumed that the best way to define malnutrition is by using the SD of the WHO-growth curves [19].

Malnutrition is thus classified as either acute or chronic:

Moderate acute malnutrition = - 2 < z-score P/A < -3

Severe acute malnutrition = -z-score P/A < -3

Moderate chronic malnutrition: <-2 z-score height/age < -3

Severe chronic malnutrition z-score height/age < -3

Puntis [24] has proposed criteria for nutritional intervention based on anthropometric values integrated with the patient’s clinical history, as follows:

(1) Growth or inadequate weight gain for a period > 1 month in children <2 years

(2) Weight loss or no weight gain for a period > 3 months in children > 2 years

(3) Change in weight for age down over 2 growth channels on growth charts

(4) Decrease in height velocity >2 cm/y from the preceding year during early/midpuberty

(5) Triceps skin fold < 5th percentile for age

It is possible to assess anthropometric measures during the initial medical visit, to be performed even at the primary care pediatric’s office and later, if considered necessary, to a specialist allergy clinic.

Moreover, an assessment for possible nutritional deficiency should be performed on children older than two years whose height growth rate is <4 cm/year because at least 95% of children grow more than 4 cm/year [25], as well as prepubertal children whose weight gain is <1 Kg per year because about 95% of children in a population not affected by malnutrition gain more than 1 kg/year [26].

###### 

**Keynote 2** The slowing of the growth velocity is the most sensitive early indicator of nutritional deficiency [27].

#### 

##### Step 3. Dietary intake and nutritional parameters

The estimated intake of introduced nutrients can be assessed through the use of a Food Frequency Questionnaire (FFQ). The FFQ is the most common dietary assessment tool used in large epidemiologic studies of diet and health. The self-administered FFQ booklet asks participants to report the frequency of consumption and portion size of approximately 116 m line items over a defined period of time (e.g. the last month; the last three months). Each line item is defined by a series of foods or beverages and the portions can be indicated from pictures and serving quantities shown in the questionnaire booklet [28].

The FFQ, however, is not an accurate tool because it provides qualitative and only semi-quantitative type information. The 3-day food diary, which consists of recording the amount and type of foods and drinks consumed by the child over a period of three consecutive days, including 2 weekday and 1 weekend days, represents the most accurate tool for children over six months especially underwent to nutritional intervention because it provides quantitative type information [29].

The results obtained from the daily food diary nutrition should be compared with the Recommended Dietary Intake (RDA) [30].

To complete a nutritional assessment, when malnutrition is suspected according to the criteria defined above, a number of laboratory bio-markers of nutritional status should be performed:

– Blood count

– Electrolytes

– Azotemia, Creatinine

– Lipid profile (total cholesterol, HDL, LDL, triglycerides)

– Protein profile (protein electrophoresis, albumin, prealbumin, RBP)

– Iron status indicators (serum iron, ferritin, transferrin)

– Plasma assay of other nutritional factors (e.g. vitamins, zinc)

The dosage of total protein has a limited value. In contrast, some plasma proteins reflect synthetic function and can be used as markers of nutritional status (see Table 1).

**Table 1:** Main plasma proteins used as markers of nutritional status

**Plasma protein**	**Distribution volume and half-life range**	**Plasma levels**
Albumin	Distribution volume: large	3,6-4,5 g/dl
	Half-life range: 15–20 days	At birth 80% of adult value
Thyroxin-binding-pre-albumin	Distribution volume: small	17,6-36 mg/dl
	Half-life range: 2–3 days	At birth: 80% of adult value
Retinol-binding protein (RBP)	Distribution volume: small	60 ± 16 mg/l
	Half-life range: 12 hours	0–10 years, 60% of adult value

Proteins with a small pool and a short half-life range should be preferred since it is a more sensitive indicator of the synthesis and protein catabolism [31]. The thyroxin-binding-prealbumin and retinol binding protein combination is an early and sensitive index of malnutrition and can be used to monitor the efficacy of renutrition. Moreover, instrumental investigations may be used for the evaluation of the catabolism of body mass, including the axial X-ray densitometry (DEXA), of proven effectiveness [32].

#### 

##### Step 4. Nutritional intervention and nutrients needs

**Energy and proteins** Children with food allergies, especially if multiple, are at risk of reduced protein-calorie intake [5-7]. The protein-calorie intake will depend on the number of diagnosed allergies.

In general, the number of meals throughout the day, the number of food items consumed, the caloric density of the food and the portions size influence the caloric intake. Infants possess an innate ability to self-regulate caloric intake (e.g. they consume larger portions than average if the frequency of meals is reduced) [33]. However, the ability of self-regulate can be influenced by factors, such as the food taste, which alter the eating behavior overpowered by the sense of hunger [34].

On the other hand, an adequate caloric intake is essential for allergic children. If this is not the case, the free amino acids are oxidized to produce energy and become useless for the purpose of protein synthesis [30].

The protein sources of high biological value include primary allergens, including milk, eggs, soy, fish and nuts. For this reason, planned diets should ensure an adequate intake of essential amino acids through the complementation of alternative protein sources, such as legumes and vegetables.

Particular attention should be given to children who have to eliminate two or more sources of animal protein from their diet, making it essential to resort almost exclusively to proteins of plant origin.

The latter have a bioavailability 10-20% lower compared to animal proteins, so from a practical standpoint, the recommendation will be to increase protein intake by 20% from 2 to 6 years, and by 15-20% in children over six years [35].

Use of protein hydrolysates or amino acid-based formulas may be necessary for children older than 1 year to ensure an adequate protein intake.

The energy and protein demands of children with FA could be increased in children suffering from moderate to severe atopic dermatitis and food allergies with gastrointestinal symptoms. In such cases, to improve the nutritional status, children should be provided with a caloric proteic intake greater than the recommended values to obtain a recovery in growth.

In 2007, WHO/FAO/UNU [36] published guidelines on the energy and protein requirements addressing catch-up growth of malnourished children. According to these guidelines, the ideal ratio of protein/energy in malnourished children varies between 8.9% and 11.5%, ration which ensures the evidence of about 70% of lean body mass and 30% of body fat (see Table 2).

**Table 2:** **Energy and protein required to achieve different rates of catch-up growth**[36]

**Catch-up growth (g/kg/day)**	**Protein (g/kg/day)**	**Energy (kcal/kg/day)**	**Protein/energy (%)**
1 g/kg/day	1,02	89	4,6
2 g/kg/day	1,22	93	5,2
5 g/kg/day	1,82	105	6,9
10 g/kg/day	2,82	126	8,9
20 g/kg/day	4,82	167	11,5

##### 

**Keynote 3** Nutrition intervention depends on the subject’s nutritional status at the time of the diagnosis, the number of diagnosed allergies, as well as the concurrent presence of atopic eczema and gastrointestinal disorders.

### Lipids

The lipid intake may be inadequate in subjects on exclusion diet, both in terms of qualitative and total intake. This issue needs careful consideration not only due to the resulting restriction of caloric intake but also for the possible lack of essential fatty acids.

The fatty acids plasma profile observed in allergic children on exclusion diet [37] leads to the assumption that not only there is a reduced intake, but also a probable consumption of long-chain omega-3 polyunsaturated fatty acids within the allergic inflammation.

To provide monounsaturated fatty acids, polyunsaturated fats and essential fatty acids in suitable quantities [27], the use of vegetable oils and olive oil is recommended in subjects with food allergies. The amount of the oils requested in the diet should be individualized on the basis of the number of foods allowed and the daily amount that is consumed.

This approach ensures an adequate intake of essential fatty acids and thus promotes an adequate growth [38].

Supplementation through algal-derived products should be considered on an individual basis.

### Carbohydrates

Even in allergic children following an exclusion diet, glycemic index carbohydrates account for 90-95% of the total energy coming from the intake of carbohydrates in the diet. Cereals, fruits and vegetables are a good source of carbohydrates.

The range of recommended intake of carbohydrates varies between 40-60% of the individual’s total daily calories [39].

The consumption of carbohydrates should be stressed also in allergic children not only because of the nutritional contribution, but also for the fiber it provides. Although cooked fruits and vegetables may not provide the same amount of nutrients compared to the raw form, they supply the same amount of fiber.

In clinical practice, it is necessary to ensure children allergic to wheat an adequate intake of carbohydrates, which are an essential energy source for proper brain function, from alternative sources in order to prevent a ketosis state.

### Micronutrients

Micronutrients include vitamins, minerals and trace elements. Generally, a varied diet contributes to the introduction of adequate amounts of all nutrients, both macro and micro. Table 3 shows which vitamins and minerals may be reduced by specific restrictions. For example, for children who avoid cow’s milk and all dairy products, vitamins and minerals found in this food group will need to be supplied from other sources [27].

**Table 3:** Vitamins and minerals contained in key foods

**Food**	**Vitamins and minerals**
Milk	Vitamin A, vitamin D, riboflavin, pantothenic acid, vitamin B12, calcium, phosphorus
Eggs	Riboflavin, pantothenic acid, vitamin B12, biotin, selenium
Soy	Thiamine, riboflavin, pyridoxine, folate, calcium, phosphorus, magnesium, iron, zinc
Grain	Thiamine, riboflavin, niacin, iron, folate, if fortified
Peanuts	Vitamin E, niacin, magnesium, manganese, chromium
Fish	Zinc, iron-heme

#### 

**Keynote 4** Pharmacological supplementation should be considered when dietary modifications are inadequate to meet vitamin, mineral, and trace element needs.

### Allergy to cow's milk protein

Cow’s milk allergy (CMA) is common in childhood and needs specific dietary interventions [40]. Cow’s milk is an important source of calcium, phosphorus, vitamin B2 (riboflavin), B5 (pantothenic acid), B12 (cobalamin), D, proteins and lipids. A reduced intake of these nutrients may be caused by a cow's milk-free diet [6,8].

Breastfeeding is recommended for infants with any type of food allergy. If the infant is allergic to milk, it may be helpful to eliminate cow’s milk protein from the mother’s diet [2].

Choosing the right formula for infants allergic to cow’s milk is still a matter of debate. Generally, the formula should be based on clinical conditions, age, nutritional characteristics of the special formula, residual allergenicity of the formula, palatability and in some social context, also on the cost.

An exhaustive and detailed discussion on which hypoallergenic formulas should be administered in different clinical situations is out of the scope of the present document and it is provided by guidelines for managing CMA in infants [2,41-43].

Foodstuffs such as almond milk or other alternative milk beverages are not suitable to the nutritional needs of children and, therefore, they should not be used as cow’s milk substitutes.

Several studies describe malnutrition, including severe cases due to improper use of the over the counter foodstuff considered safe [44,45].

With regard to the intake of mammalian milks during the first year of life, donkey’s milk has a proteomic profile similar to human milk [46] and it has been demonstrated well tolerated in clinical studies performed on cow’s milk allergic children aged 12 months or above [47,48].

Nevertheless, donkey’s milk has a low fat content that corresponds to a low energetic value [49]. This limits its use for infants on exclusively milk diet [50].

To this purpose, there are no well-designed clinical studies evaluating the nutritional efficiency of donkey’s milk in the first year of life. The few data are limited to a selected case-report series [51], to the use of modified donkey’s milk by adding medium-chain triglycerides or sunflower oil in a retrospective study [52] or to small-sample study [53]. All of these drawbacks call in question the validity of the results of these studies.

Therefore, donkey’s milk should not be recommended as a suitable alternative to infants on exclusive-milk diet. It could be considered for infants on a solid-foods diet or after the first year of life on an individual basis.

Goat’s milk is not suitable for consumption by infants and children with CMA, given the high protein content, excessive osmolarity and low content of vitamin B12 and folic acid [54] besides the high risk of cross-reactivity, up to 90 percent of cow’s milk allergic infants [55].

Formula substitute should be continued until the child develops tolerance. However, if tolerance is not reached, administration should be continued until at least the age of 2 and if needed, even further to increase the nutritional and protein intake [27].

An inadequate intake of calcium was observed in subjects following a diet without protein from cow's milk [40], even in those who received infant formula [6].

Supplementation of calcium salts at doses of 500 to 1000 mg/day in randomized controlled trials has shown positive effects on bone health in terms of bone mineral density in subjects during prepuberty [56-58].

None of these studies have reported adverse events.

An increased risk of fractures was reported in children and adolescents following a cow’s milk-free diet with inadequate calcium supplementation [59,60].

In the first six months of life, a supplementation with calcium, the type of milk substitute (see Table 4), and the infant’s daily intake should be assessed from time to time.

**Table 4:** Calcium and vitamin D content in alternative formulas

**Formula**	**Ca**	**Vit D**
	**mg/100 kcal**	**mg/100 kcal**
Extensively hydrolysed whey	61–93	1,49–2,06
Extensively hydrolysed casein	113–138	1,47–1,84
Follow-up soy	90–97	1,43–2,06
Follow-on hydrolysed rice protein	69–89	1,18–1,55
Follow-up hydrolysed rice protein	106–120	1,69–2,54
Aminoacid based formula	94–98	1,25–1,79

In the second semester of life, solid foods are introduced and the consumption of formula progressively decreases. When the intake of milk falls below 500 ml/day supplementation is required.

In children with CMA who do not reach tolerance, supplementation with calcium is also recommended after the first year for the entire duration of the exclusion diet.

The supplementary dose of elemental calcium can vary from 500 mg/day during the early years of life to 1000 mg/day or more during adolescence, remaining below the maximum tolerable dose (UL) [61] according to the recommended intake per age [62] (see Table 5).

**Table 5:** Calcium and vitamin D dietary reference intakes by life stage IOM 2011

	**Calcium**		**Vitamin D**	
**Life stage group**	RDA** (mg/day)	UL* (mg/day)	**RDA (mg/day)**	**UL (IU/day)**
**(age and gender)**				
1–3 yr (M + F)	700	2500	600	2500
4–8 yr (M + F)	1000	2500	600	3000
9–13 yr (M + F)	1300	3000	600	4000
14–18 yr (M + F)	1300	3000	600	4000
Infants				
0–6 months (M + F)	200	1000	400	1000
6–12 months (M + F)	260	1500	400	1500

M, Male; F, female.

*UL indicates level above which there is risk of adverse events. The UL is not intended as a target intake (no consistent evidence of greater benefit at intake levels above RDA).

**RDA: Recommended Dietary Allowances; intake that covers needs of ≥97.5% of population.

The maximum supplementary dose of elemental calcium should be less than or equal to 500 mg/day at a time [63].

Calcium carbonate and calcium citrate are the most commonly used supplements. They differ for the concentration of elemental calcium. Calcium carbonate contain 40% elemental calcium, whereas calcium citrate supplements contain only 21% elemental calcium [64]. Therefore, it is needed to use more tablets of calcium citrate to obtain the desirable dose of elemental calcium.

When calcium is taken with meals, absorption equal to about 30% and is similar for both preparations [65].

Calcium supplementation should always be combined with vitamin D supplementation since the absorption of calcium contained in food decreases from 30-40% to 10-15% when a deficiency of vitamin D exists [66].

During the first year of life, supplementation with vitamin D should be administered as in the general pediatric population. Afterwards, it is appropriate to continue the supplementation with vitamin D throughout the entire period of the exclusion diet, although it is currently unknown what dose to use. However, in view of the recognized role of vitamin D in regulating the immune system, levels higher than RDA but lower than the Upper Level (UL) [61,67] may be fitting.

Supplementation with calcium and vitamin D are important in individuals with cow’s milk allergy on exclusion diet at any age, as it is difficult to meet recommended dietary allowances for calcium without concomitant consumption of dairy foods or supplements [68].

Following the European Society for Paediatric Gastroenterology Hepatology and Nutrition (ESPGHAN) [69] and European Food Safety Authority (EFSA) Recommendations [70], weaning of children allergic to milk with cow’s milk allergy should not be performed differently than non-allergic subjects.

### Allergy to other foods: wheat allergy and multiple food allergies

Wheat is rich of low glycemic index carbohydrates, vitamin B1 (thiamine), B2, B3, niacin, and iron. The wheat allergic patient must avoid all wheat containing foods, resulting in the elimination of many processed and manufactured products including bread, cereal, pasta, crackers, cookies, and cakes.

Many alternative grains are available to patients with wheat allergy, including oat, barley, buckwheat, rye, amaranth, millet, and quinoa. Twenty percent of individuals with one grain allergy may be clinically reactive to another grain [71]. Thus, use of these alternative products should be individualized and based upon tolerance as determined by the patient's allergy specialist.

In children with multiple food allergies continued use of commercially prepared complete formulas beyond infancy is recommended if sufficient intake of good-protein quality is compromised [27].

As meeting dietary needs may be difficult when multiple food allergies occur, a proper diet must be agreed between the allergist and the nutritionist case by case.

#### 

**Keynote 5** In subjects with multiple food allergies, the variety of combinations from individual allergies does not allow to formulate standard dietetic advices. An individualized approach will be agreed between the allergist and the nutritionist according to the issues that come out from the nutritional assessment.

### Follow up

An appropriate follow- up of allergic children on exclusion diet is essential.

Children’s diet vary as they get older, and nutritional requirements also change accordingly.

The periodic re-evaluation of the child is needed also to assess the compliance to the diet.

It is useful to explain to the parents when they are first seen the importance of regular visits to ensure that the diet continues to satisfy the nutritional needs of the child.

The follow-up visits should be established on the basis of the age of the child and following the growth pattern, according to the following plan [72]:

– In the first semester of life: monitor weight, length and dietary compliance at the age of 1, 2 and 4 months;

– In the second semester of life: evaluate anthropometric parameters (weight, length) and assess the dietary compliance on a quarterly basis (6, 9 and 12 months).

– After the first year: evaluate growth rate every 6–12 months.

– Assessment of the nutritional intake: at least once a year with regular growth; otherwise, two or more times per year when there is alteration of the normal growth pattern.

Nutritional and anthropometrical follow-up may be best provided through a continuing relationship between the family pediatricians (primary care level) and the professional team of the secondary and tertiary levels of care.

## Conclusions

In children with food allergies, exclusion diet may cause nutritional and growth deficiency; therefore, it needs to be tailored to each individual and periodically re-evaluated.

A multidisciplinary approach, which includes the interaction of the nutritionist, dietician, nurses, allergologist and whenever possible psychologist, is the most successful way to ensure both growth and health of allergic children and to help family members to deal with the daily challenges in which they are involved.

Future challenges include definition of several unmet needs. They are as follows:

– Elucidation of the mechanisms by which restricted diet and atopic illnesses induce growth impairment.

– Identification of biomarkers that are predictive of poor growth in children with allergy and atopic diseases.

– Definition of the real nutritional needs of the allergic children

– Standardization of pharmacological supplementation as suitable surrogate of the food excluded from the diet.

– Evaluation and confirmation of the dietetic regimen associated with better clinical outcomes

– Development of methods for identifying diet-responsive and nonresponsive phenotypes.

– Well-designed long-term study to assess the effectiveness of supplementation and food substitute and adherence to the diet in real life.

## Abbreviations

FA: Food allergy; SINUPE: Italian Society of Pediatric Nutrition; SIAIP: Italian Society of Pediatric Allergy and Immunology; BMI: Body mass index; WHO: World Health Organization; CDC: Centers for diseases controls and prevention; SD: Standard deviation; FFQ: Food frequency questionnaire; RDA: Recommended dietary intake; DEXA: Axial X-ray densitometry; CMA: Cow’s milk allergy; UL: Upper level; ESPGHAN: European Society of Paediatric Gastroenterology Hepatology and Nnutrition; EFSA: European Food Safety Authority.

## Competing interests

The authors declare that they have no competing interests.

## Authors’ contributions

MG, FG. ED’A, AB. CC, AB. EV, ES. SB, ES. LI, ES. IDI, AB. AM, AB. ER, FG. RB, ES. All authors read and approved the final manuscript.
